# Small bowel adenocarcinoma incarcerated within an inguinal hernia

**DOI:** 10.1186/s40792-019-0765-8

**Published:** 2019-12-27

**Authors:** Hironari Kawai, Koichiro Haruki, Naoki Takada, Toshio Iino, Masahiro Ikegami, Katsuhiko Yanaga

**Affiliations:** 1 Department of Surgery, The Saitama Jikei Hospital, 3-208, Ishihara, Kumagaya, Saitama 360-0816 Japan; 2 Department of Surgery, The Jikei University School of Medicine, 3-25-8, Nishi-Shimbashi, Minato-ku, Tokyo, 105-8461 Japan; 3 Department of Pathology, The Jikei University School of Medicine, 3-25-8, Nishi-Shimbashi, Minato-ku, Tokyo, 105-8461 Japan

**Keywords:** Small bowel cancer, Small intestinal adenocarcinoma, Incarceration

## Abstract

**Background:**

Small bowel adenocarcinomas are rare malignant tumors that account for less than 2% of gastrointestinal malignancies. In addition, tumor incarceration in an inguinal hernia is also rare entity. We herein report a first case of small bowel adenocarcinoma incarcerated within an inguinal hernia.

**Case presentation:**

A 75-year-old man with asymptomatic anemia (hemoglobin, 8.6 g/dl) had a checkup at our hospital. Colonoscopy revealed bleeding through the ileocecal valve and an annular stricture by a tumor in the ileum. Endoscopic biopsy revealed a well-differentiated adenocarcinoma of the small bowel. Enhanced computed tomography showed a hypervascular solid tumor incarcerated within a right inguinal hernia. With a diagnosis of small bowel adenocarcinoma incarcerated within a right inguinal hernia, the patient underwent elective laparotomy with midline excision. The small bowel tumor, located at 30 cm from the terminal ileum, was incarcerated within a right inguinal hernia, and the small bowel was adherent to the hernia sac. A 24-cm segment of the distal ileum and regional lymph nodes were resected. The hernia sac was ligated, and the bottom of the hernia sac was resected. The hernia orifice was closed by tissue repair technique via a standard oblique incision in the right inguinal region. Postoperatively, the patient remains well with no evidence of tumor or hernia recurrence as of 1 year after operation.

**Conclusions:**

We reported to our knowledge the first case of small bowel adenocarcinoma incarcerated within an inguinal hernia.

## Background

Adenocarcinoma of the small bowel is rare and accounts for less than 2% of gastrointestinal malignancies, which is usually detected by video capsule endoscopy [1]. Tumor incarceration within an inguinal hernia is also rare with an estimated incidence of < 0.5%, which is usually found during emergency operation for the incarcerated hernia. We herein report a first case of small bowel adenocarcinoma incarcerated within an inguinal hernia, which was successfully diagnosed preoperatively by colonoscopy and computed tomography (CT).

## Case presentation

**Fig. 1:**
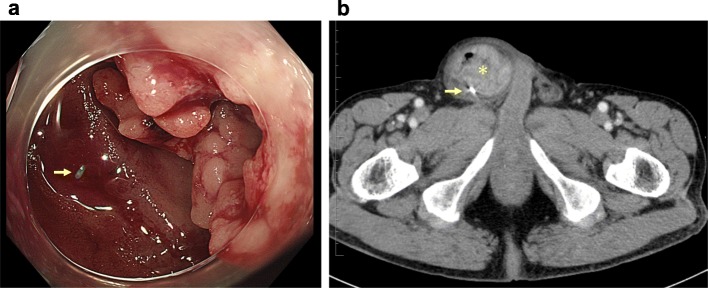
**a** Colonoscopy revealed bleeding through the ileocecal valve. Therefore, we advanced the scope into the ileum and found an annular stricture by a tumor, for which endoscopic biopsy and clip-marking (arrow) of the tumor were performed. **b** Enhanced CT demonstrated a hypervascular solid tumor (asterisk) and a metal clip (arrow) by colonoscopy in the right inguinal hernia

**Fig. 2:**
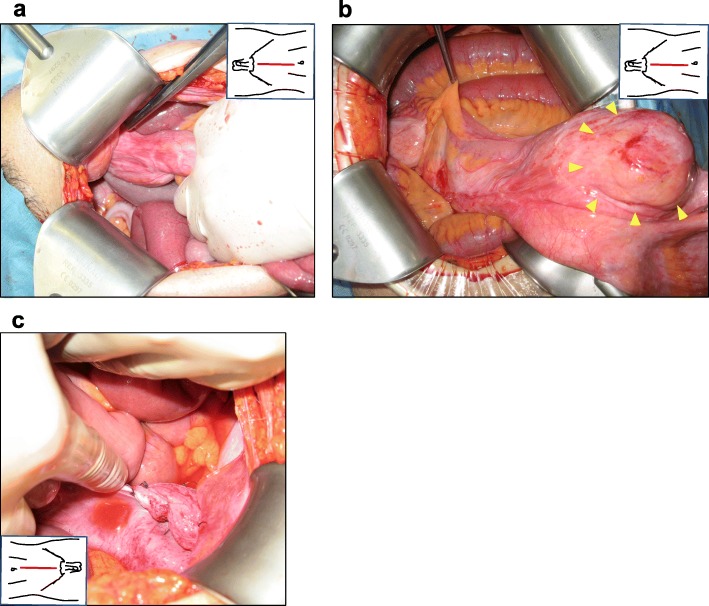
**a** The small bowel tumor, located at 30 cm from the ileum end, was incarcerated within a right inguinal hernia. **b** After adhesiolysis, the tumor (arrowheads) was reduced into the peritoneal cavity. **c** The reduced hernia sac was ligated, and the bottom of the hernia sac was resected from the peritoneal cavity side

**Fig. 3:**
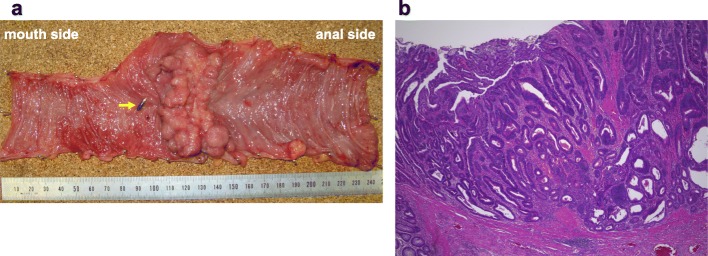
**a** The excised specimen revealed a type V (unclassifiable type) tumor with the diameter of 70 × 40 mm with nodule-aggregating lesions and a localized ulcer. The marking clip (arrow) was located at 1 cm distal of the tumor. **b** Pathological examination revealed well-differentiated adenocarcinoma of the small bowel with no lymph node metastasis

A 75-year-old man with asymptomatic anemia (hemoglobin, 8.6 g/dl) had a checkup at our hospital. The patient had no significant past medical history, family history, or predisposing conditions such as familial adenomatous polyposis, hereditary non-polyposis colorectal cancer, Peutz-Jeghers syndrome, and Crohn’s disease. The patient was noted to have a non-reducible right inguinoscrotal hernia on examination. The serum carcinoembryonic antigen and carbohydrate 19-9 were within normal range. Colonoscopy revealed bleeding through the ileocecal valve, and therefore, we advanced the scope into the ileum and found an annular stricture by a tumor, for which endoscopic biopsy and clip-marking of the tumor were performed (Fig. 1a). The biopsy revealed well-differentiated adenocarcinoma. Enhanced CT showed a hypervascular solid tumor and a metal clip in the right inguinal hernia (Fig. 1b). With a diagnosis of small bowel adenocarcinoma incarcerated within a right inguinal hernia, the patient underwent elective laparotomy with midline excision. There was no metastatic lesion including liver metastasis and peritoneal dissemination. The small bowel tumor, located at 30 cm from the terminal ileum, was incarcerated within a right inguinal hernia, and the small bowel was adherent to the hernia sac although there was no evidence of direct invasion into the hernia sac (Fig. 2a). After adhesiolysis, the tumor and the hernia sac were reduced into the peritoneal cavity (Fig. 2b). A 24-cm segment of the distal ileum and regional lymph nodes were resected, and triangle anastomosis was performed for reconstruction. The reduced hernia sac was ligated, and the bottom of the hernia sac was resected from the peritoneal cavity side (Fig. 2c). Finally, the hernia orifice was closed by tissue repair technique via a standard oblique incision in the right inguinal region. To avoid possible infection, we chose a tissue repair technique without a mesh. The excised specimen revealed type V (unclassifiable type) tumor with the diameter of 70 × 40 mm with nodule-aggregating lesions and a localized ulcer (Fig. 3a). Pathological examination revealed well-differentiated adenocarcinoma of the small bowel with no lymph node metastasis [Union International Control Cancer classification of malignant tumors 7th edition; pT2, pN0, pM0, stage I, R0] (Fig. 3b). Postoperatively, the patient was discharged on postoperative day 8 without complications. The patient did not receive adjuvant therapy and remains well with no evidence of tumor or hernia recurrence as of 1 year after operation.

## Discussion

This is the first case report of small bowel adenocarcinoma incarcerated within an inguinal hernia in the English literature. Adenocarcinoma of the small bowel is rare and accounts for less than 2% of gastrointestinal malignancies, and tumor incarceration within an inguinal hernia is also rare with an estimated incidence of < 0.5%; therefore, small bowel adenocarcinoma incarcerated within an inguinal hernia is extremely rare [1, 2]. Furthermore, the present case has some noteworthy points. First, small bowel adenocarcinoma within an inguinal hernia was successfully diagnosed preoperatively at stage I. Since most small bowel cancers remain asymptomatic until advanced, the majority of patients are diagnosed at advanced stages (32% stage IV, 27% stage III, 30% stage II, and 10% stage I) [3]. Besides, Kanemura et al. reviewed 31 cases of colon carcinoma incarcerated within an inguinal hernia and reported only a half of the cases were diagnosed preoperatively, while the other half were diagnosed incidentally during emergency operation for incarcerated hernia [4]. Therefore, small bowel adenocarcinoma incarcerated within an inguinal hernia which was preoperatively diagnosed at stage I was rare condition. Second, the tumor was located in the ileum which is a rather rare site for small bowel adenocarcinoma. Although small bowel adenocarcinomas are found throughout the length of the small bowel, more than half (56%) are located in the duodenum, while tumors of the jejunum and ileum represent 30% and 20%, respectively [5]. Therefore, tumor location itself was relatively rare. Finally, small bowel adenocarcinoma within an inguinal hernia was detected by computed tomography and colonoscopy instead of video capsule endoscopy and upper endoscopy which are usually needed as screening tools for detecting small bowel cancers.

Taken together, the condition that small bowel adenocarcinoma incarcerated within an inguinal hernia was successfully diagnosed preoperatively at stage I and was detected by computed tomography and colonoscopy was extremely rare. The patient neither has clinical symptoms of small bowel adenocarcinoma except for anemia, has family history, nor has predisposing conditions; therefore, preoperative diagnosis of adenocarcinoma of the small bowel was challenging in the current setting [6, 7]. However, we could detect the tumor preoperatively by performing the colonoscopy for anemia assessment and could perform the definitive cancer surgery and hernia repair. In the case that patients have long-standing incarcerated hernia and anemia, exploration of the terminal ileum by colonoscopy may help to detect small bowel adenocarcinomas which are located in the terminal ileum. By detecting the tumor incarceration within an inguinal hernia in advance, it might be easier to select the appropriate approach for definitive cancer surgery and hernia repair.

## Conclusions

We reported to our knowledge the first case of small bowel adenocarcinoma incarcerated within an inguinal hernia.

## Data Availability

The dataset supporting the conclusion of this article is included within the article.

## Abbreviations

CT: Computed tomography
